# Organic Sunscreens—Biological Activity from an Enzymatic Perspective

**DOI:** 10.3390/molecules31101656

**Published:** 2026-05-14

**Authors:** Anna W. Sobańska, Andrzej M. Sobański, Elżbieta Brzezińska

**Affiliations:** 1 Department of Analytical Chemistry, Medical University of Lodz, 90-151 Łódź, Poland; elzbieta.brzezinska@umed.lodz.pl; 2 Faculty of Chemistry, University of Lodz, 91-403 Łódź, Poland; andrzej.sobanski@edu.uni.lodz.pl

**Keywords:** organic sunscreens, biological activity, butyrylcholinesterase inhibition

## Abstract

Selected organic sunscreens from different chemical families were investigated in the context of their ability to inhibit butyrylcholinesterase using novel Multiple Linear Regression, Artificial Neural Network, and Support Vector Regression models based on a set of six independent variables commonly associated with compounds’ absorption and distribution properties. It was established that the descriptors that have a particularly strong, positive influence on the ability of compounds to inhibit BChE expressed as p***IC***_50_ are the count of rotatable bonds (***nRot***) and lipophilicity (log***D***); p***IC***_50_ is negatively correlated with flexibility (***Flex***), fraction of sp^3^ carbon atoms (***F***_sp3_), caco-2 permeability (***caco2***), and plasma protein binding ability (***PPB***). The sunscreens that are likely to be particularly strong BChE inhibitors are Ethylhexyl Triazone (ET), Diethylhexyl Butamido Triazone (DOBT), Octocrylene (OCR), and Diethylamino Hydroxybenzoyl Hexyl Benzoate (DHHB). However, it must be stressed that ET and DOBT lie outside the chemical space of the reference compounds, so predictions for these two compounds should be treated with caution.

## 1. Introduction

Organic sunscreens are used in personal care products to protect skin and hair from harmful ultraviolet radiation, and in other products (textiles, dyes, household chemical products) to prevent photodegradation by converting UV radiation into thermal energy upon absorption [1]. It has been demonstrated that sunscreens help prevent sunburn, solar keratosis, and non-melanoma skin cancers [2]. Although organic sunscreens are considered beneficial when used reasonably [3], some compounds in this group are biologically active—they cross biological barriers and are known or suspected endocrine disruptors, i.e., compounds that interfere with human and animal hormonal systems and are thus likely to impair developmental, reproductive, neurological, and immune functions [4,5]. Several in vitro and in vivo studies (mainly on animal models, Table 1) demonstrated that organic sunscreens (especially benzophenone-3; according to some studies also benzophenone-2, homosalate, OD-PABA, PABA, 3-benzylidene camphor, 3-(4-methyl-benzylidene) camphor, 2-ethylhexyl 4-methoxy cinnamate, octocrylene) affect estrogenic signaling, exhibit antiandrogenic and progesterone activity, induce changes in weight and histology of reproductive organs in both sexes, and interfere with the hypothalamic–pituitary–thyroid axis [2,6,7,8].

Humans are exposed to organic sunscreens through direct contact (skin absorption, ingestion, and inhalation) and indirect routes (contaminated water and seafood) [5], with detectable levels of organic sunscreens found in urinary samples from the vast majority of the US population [6]. Organic sunscreens were detected in drinking water supplies in countries such as Spain, Australia, Singapore, and Brazil [9].

Studies indicate a negative correlation between exposure to organic UV filters and adiposity measures in peripubertal boys, but not in girls [10]; maternal exposure to organic sunscreen during pregnancy alters offspring birth outcomes [10].

Sunscreens are primarily metabolized in the human liver (a detailed report on the hepatic metabolism of oxybenzone, avobenzone, octocrylene, octinoxate, octisalate, and homosalate was provided in [9]) and in other organs, such as the skin [11]. The metabolic pathways of sunscreens are complex, involving processes such as ester hydrolysis (if applicable), oxidation, and covalent binding to glutathione [12]. Some toxic effects of organic sunscreens may also be attributed to their metabolites, which appear to be more reactive electrophiles than the parent compounds. For example, skin metabolism of certain benzophenone sunscreens (benzophenone-3 and dioxybenzone) may yield potentially phototoxic glucuronide metabolites [13]. Organic sunscreens were also investigated for potential metabolism- and transporter-mediated interactions with co-administered drugs. In vitro, 4-methylidenecamphor and benzophenone-3 inhibit CYP2C9 and the renal transporters OAT3 and OCT2, but their ***IC***_50_ values exceed clinically relevant plasma levels [14].

Elevated bioactivity has also been reported in synergistic mixtures of organic sunscreens, which is a matter of particular concern because sun protection formulations typically contain multiple UV filters [7,15,16]. However, it must be stressed that some sunscreen mixtures exhibit antagonism—their toxicity is lower than that of the individual compounds [17,18,19].

Large quantities of organic sunscreens are released into the environment worldwide; the products of their environmental transformations appear to be more hazardous than the parent compounds, and some are more persistent in the environment [20,21,22].

The undesired biological activity of organic sunscreens was also studied in the context of their potential interactions with the main detoxifying enzymes in the placenta: (i) glutathione-S-transferases, which catalyze the conjugation of reduced glutathione (GSH) to various electrophiles, thereby facilitating their excretion; (ii) N-acetyltransferase 2, responsible for acetylating compounds such as aromatic amines [23]. The key human health and environmental concerns related to sunscreen exposure are presented in Table 1.

**Table 1:** The key human health and environmental concerns related to sunscreens.

Sunscreen	Main Human Health Concerns	Main Environmental Concerns	Reference
Benzophenones	Systemic absorption; endocrine, reproductive, developmental, thyroid and neurotoxic potential; detected in urine, breast milk, fetal circulation; associations with altered testosterone, thyroid hormones, sperm quality, fecundity, and birth outcomes	Widely detected in wastewater and aquatic systems; bioaccumulative; contribute to adverse effects in aquatic organisms	[24,25,26,27,28,29,30,31,32,33,34]
Octinoxate (EHMC)	Endocrine-disrupting activity (sex hormones, thyroid axis); possible reproductive toxicity	Ecological risk in marine and freshwater	[35,36,37,38,39,40]
Octocrylene (OCR)	High systemic absorption; slow elimination; internal exposure high; potential neurotoxicity; possible endocrine effects	High aquatic risk in both marine and freshwater; bioaccumulation in marine biota	[18,41,42]
Avobenzone (BMDM)	Systemic absorption; potential endocrine disruption (sex and thyroid hormones); hormonal changes reported in human/experimental studies	Freshwater risk at elevated concentrations; presence in recreational waters	[18,43,44]
Homosalate (HMS), Octisalate (OS)	Possible endocrine activity; systemic detection after use; epidemiologic links to hormonal endpoints are limited and mixed	Detected in the environment; contribute to the overall UV-filter load	[44,45,46]
MBC	Alterations in the reproductive axis in males (rat model)		[47]
Mixed organic UV filters, in particular: MBC, HMS, OS, PABA, IMC, EHDP	Systemic absorption through skin; potential endocrine (estrogenic, androgenic, thyroid), reproductive, developmental and neurotoxic effects; contact photoallergy is the best-documented direct human toxicity; exposure through breast milk	Detected in coastal waters, wastewater, sediments, and biota; bioaccumulation; coral bleaching; toxic effects on reproduction, development, genetics, and neurology in marine organisms	[15,48,49,50,51,52,53,54,55,56,57,58]

The cholinergic system is a major neurotransmitter system involved in learning and memory [59]. It encompasses the neurotransmitter acetylcholine (ACh), the enzyme that synthesizes it—choline O-acyltransferase (ChAT), the muscarinic and nicotinic receptors for the neurotransmitter, and the enzymes that hydrolyze the neurotransmitter—acetylcholinesterase (AChE) and butyrylcholinesterase (BChE) [60]. Cholinergic neurons play an important role in cognitive functions, and their degeneration is considered a major factor in the development of dementia [61]. Recently, non-neuronal cholinergic systems in the skin, cardiovascular system, and placenta [62,63,64], as well as functions of the cholinergic system not directly related to neurotransmission [59,60,65], have attracted considerable attention. However, the main focus is on the CNS cholinesterase inhibitors in two contexts: (i) therapeutic—cholinesterase inhibitors used to treat symptoms of neurodegenerative diseases [66]; (ii) toxic—irreversible or reversible ACh inhibitors (mainly from the chemical families of organophosphates and carbamates) are used as pesticides (insecticides) [67]; some organophosphates are potent nerve agents [68]. New developments focus mainly on novel or repurposed drugs against Alzheimer’s disease [69,70,71,72,73,74,75]; there is also some interest in novel insecticides from chemical families other than organophosphates or carbamates [76,77].

Alzheimer’s disease is a multifactorial neurodegenerative condition, so attention often turns to drugs that inhibit cholinesterase activity and simultaneously interfere with other neurodegenerative pathways: Aβ aggregation, oxidative stress, metal dyshomeostasis, and neuroinflammation—either by targeting both the catalytic and peripheral domains of ChEs (dual-site ligands), or by acting simultaneously on cholinesterases and other targets (multi-target-directed ligands) [78,79,80].

Another enzyme involved in the cholinergic system is butyrylcholinesterase (BChE). This enzyme has long been underestimated because it was thought to be involved in no specific physiological processes; its inhibition alone seldom causes acute symptoms, and both humans and animals deprived of BChE can lead almost normal lives, although they are likely to gain weight faster on high-fat diets than individuals with normal BChE blood levels. Animal studies have shown that BChE hydrolyzes the hormone ghrelin and regulates its levels in the peripheral circulation, thereby affecting the physiological processes in which ghrelin is involved (including insulin release, fat metabolism, and adiposity) [81,82]. At present, BChE is being extensively investigated in the context of dementia pharmacotherapy [83,84,85] (many AChE inhibitors also interfere with BChE activity [86]). BChE is also considered a bioscavenger that protects AChE at nerve synapses against inhibition by toxic compounds, e.g., nerve agents from the organophosphate chemical family [87,88,89,90], and is associated with altered metabolism of some drugs [91,92,93].

The potential interactions of organic sunscreens with biological targets in the cholinergic system have received relatively little attention to date. Given the widespread use of these compounds in cosmetics, their environmental abundance, and ability to cross biological barriers, the risk of human exposure is considerable, even among individuals unaware of their use. For now, organic sunscreens are considered an environmental rather than a human health issue; if used appropriately, their benefits appear to outweigh the risks. However, their widespread presence in the environment (including drinking water resources, wildlife, and the human food chain [94,95,96,97,98,99,100,101]) is making them a global concern. In this study, we sought to address the knowledge gap regarding the biological activity of organic sunscreens by investigating their potential interference with BChE activity.

## 2. Results and Discussion

The ability of compounds to inhibit BChE, expressed as their half-maximal inhibitory concentration (***IC***_50_) or the enzyme inhibitory constant (***K***_i_), can be predicted in silico using published machine-learning, 3D QSAR, or SMILES-based QSAR models [83,102,103,104,105]. In this study, we sought to predict the p***IC***_50_ of selected organic sunscreens using novel models based on molecular descriptors typically associated with compound absorption and distribution in organisms.

**Figure 1:**
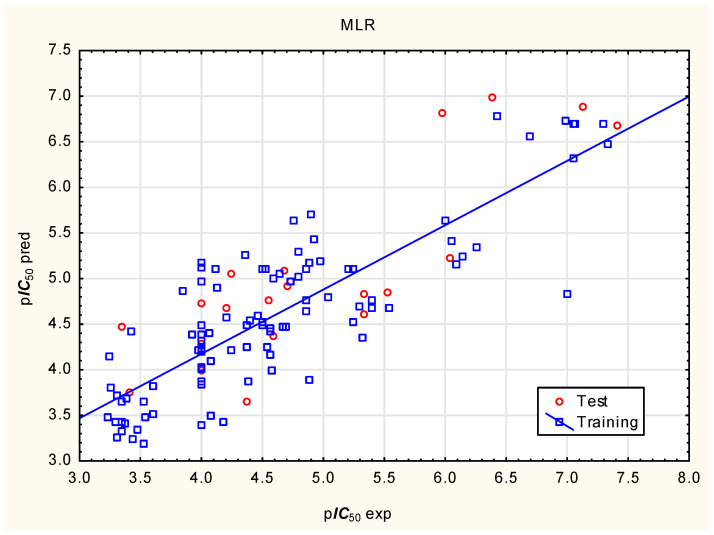
MLR model of p***IC***_50_, predicted vs. experimental values.

Based on the Multiple Linear Regression model (Equation (1), Figure 1, Table 2), the p***IC***_50_ of compounds was positively correlated with the rotatable bond count (***nRot***) and lipophilicity (log***D***) and negatively correlated with flexibility (***Flex***), the fraction of sp3 carbon atoms (***Fsp3***), Caco-2 permeability (***caco2***), and plasma protein binding ability (***PPB***). The descriptors selected for Equation (1) and used in the subsequent ANN and SVR models are sufficiently uncorrelated to justify their joint use (Table 3; tolerance > 0.25).

p***IC***_50_ = 1.68 (±1.26) + 0.481 (±0.053) ***nRot*** − 5.14 (±0.77) ***Flex*** − 1.75 (±0.47) ***F***_sp3_ + 1.18 (±0.14) log***D*** − 0.847 (±0.195) ***caco2*** − 0.0591 (±0.0113) ***PPB*** (n = 100, R^2^ = 0.706, R^2^_adj._ = 0.687, Q^2^ = 0.667, F = 37.15, *p* < 0.001, RMSE_pred_ = 0.59) (1)

**Figure 2:**
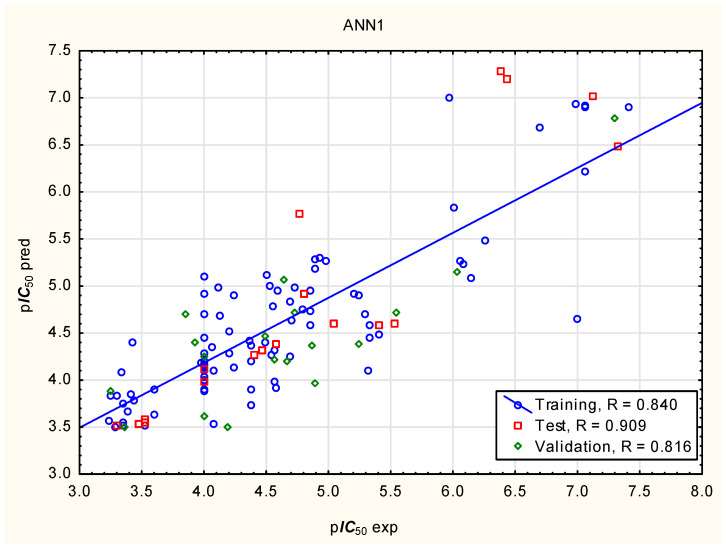
ANN model of p***IC***_50_, predicted vs. experimental values.

**Figure 3:**
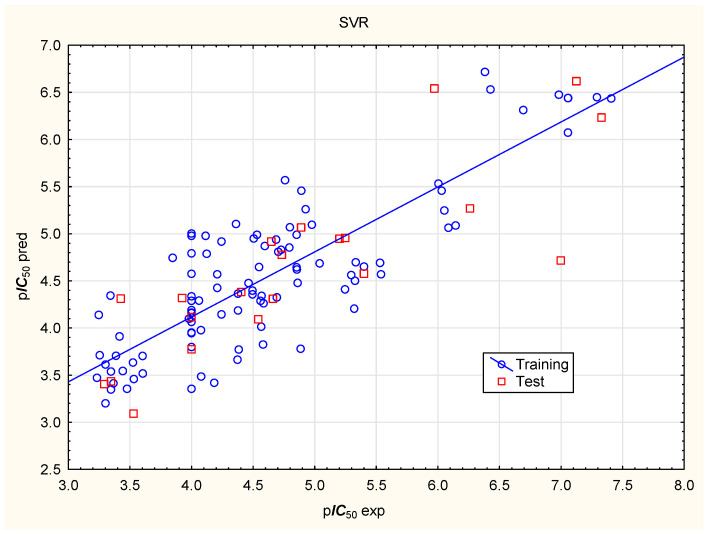
SVR model of p***IC***_50_, predicted vs. experimental values.

The same set of independent variables was used in models prepared to predict p***IC***_50_ using Artificial Neural Network (ANN) and Support Vector Regression (SVR) algorithms (Figure 2 and Figure 3; Table 4). The significance of the independent variables in the ANN models varies across models; on average, it decreases in the following order: ***Flex*** > ***nRot*** > log***D*** > ***Fsp3*** > ***PPB*** > ***caco2***, with no independent variable scoring 1 or lower in the Global Sensitivity Analysis (Supplementary Materials). The p***IC***_50_ values predicted by the MLR, ANN1, and SVR models are in close agreement (Table 2).

**Figure 4:**
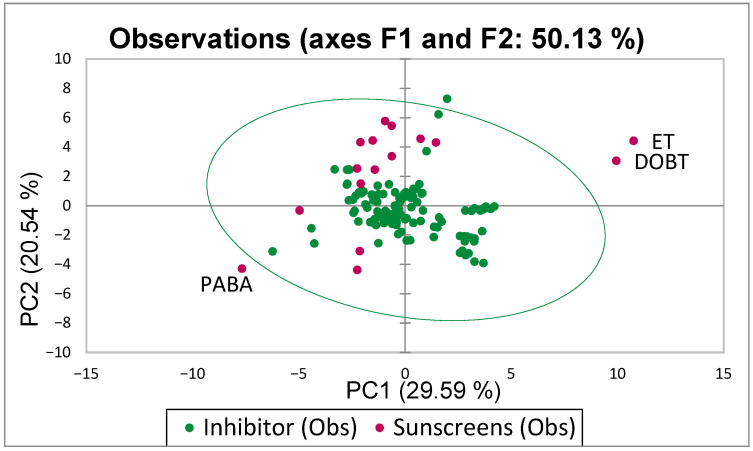
PC2 vs. PC1 for the studied sunscreens and the reference compounds.

**Figure 5:**
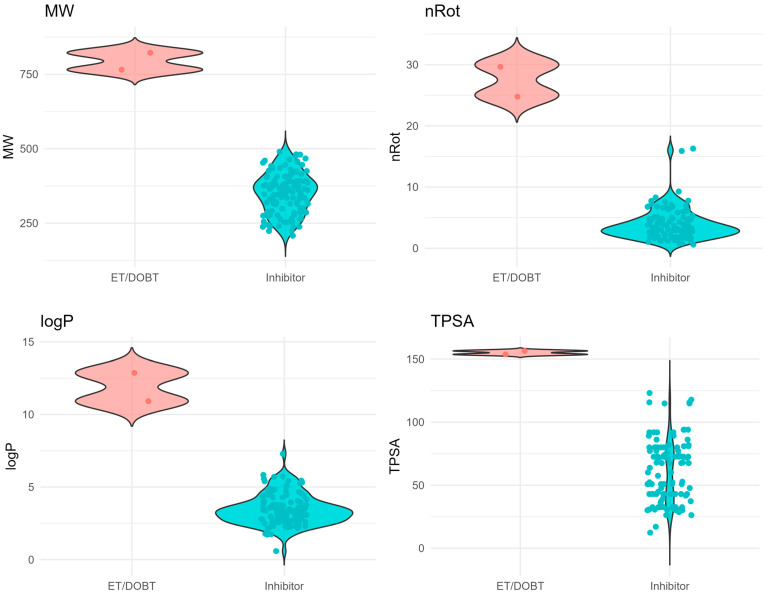
Comparison of selected physicochemical properties of reference compounds and sunscreens that fall furthest off the AD—ET and DOBT.

Based solely on the predicted p***IC***_50_ values, some sunscreens are likely to be relatively potent BChE inhibitors. Their p***IC***_50_ values are comparable to those of tacrine (IUPAC name: 1,2,3,4-tetrahydroacridin-9-amine) [106] and to those of some novel BChE inhibitors developed by Sang et al. [107], or even to those of the very strong BChE inhibitors reported by Kamal [108] or reviewed by Bubley [109]. Two compounds (ET and DOBT) have mean p***IC***_50_ values above 12 (or ca. 10–11 if results from ANN models 2 to 5 are included), indicating very high inhibitory activity. However, these compounds fall outside the chemical space defined for the reference compound group used in this study (Figure 4 and Figure 5), so predictions from the models described in Section 3.3, Section 3.4 and Section 3.5 should be treated with caution.

DHHB and OCR are also expected to be strong BChE inhibitors (p***IC***_50_ between 5 and 6). Unlike ET and DOBT, they fall within the chemical space of the reference compounds, making these results plausible. Three other sunscreens (BMDM, BP-3, PABA) have predicted p***IC***_50_ values between 4 and 5, comparable to those of donepezil, rivastigmine, or galantamine (p***IC***_50_ = 5.26, 5.5, and 4.86, respectively) [61]; the remaining sunscreens are likely to be moderate inhibitors with p***IC***_50_ values between 2 and 4.

**Figure 6:**
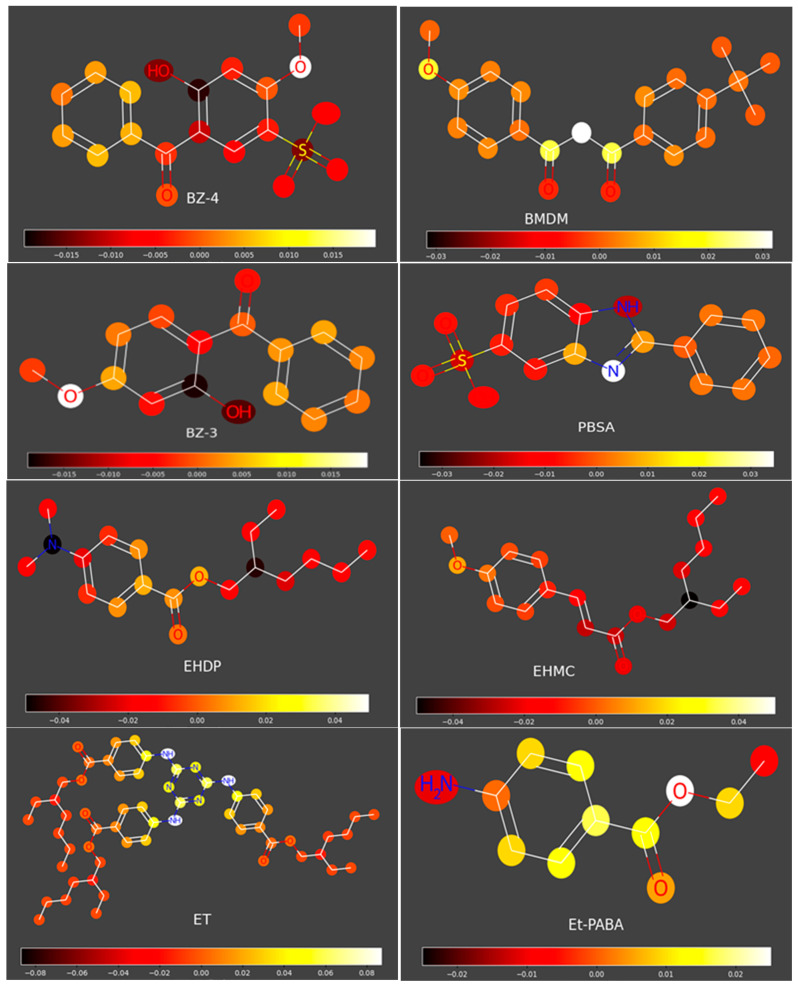

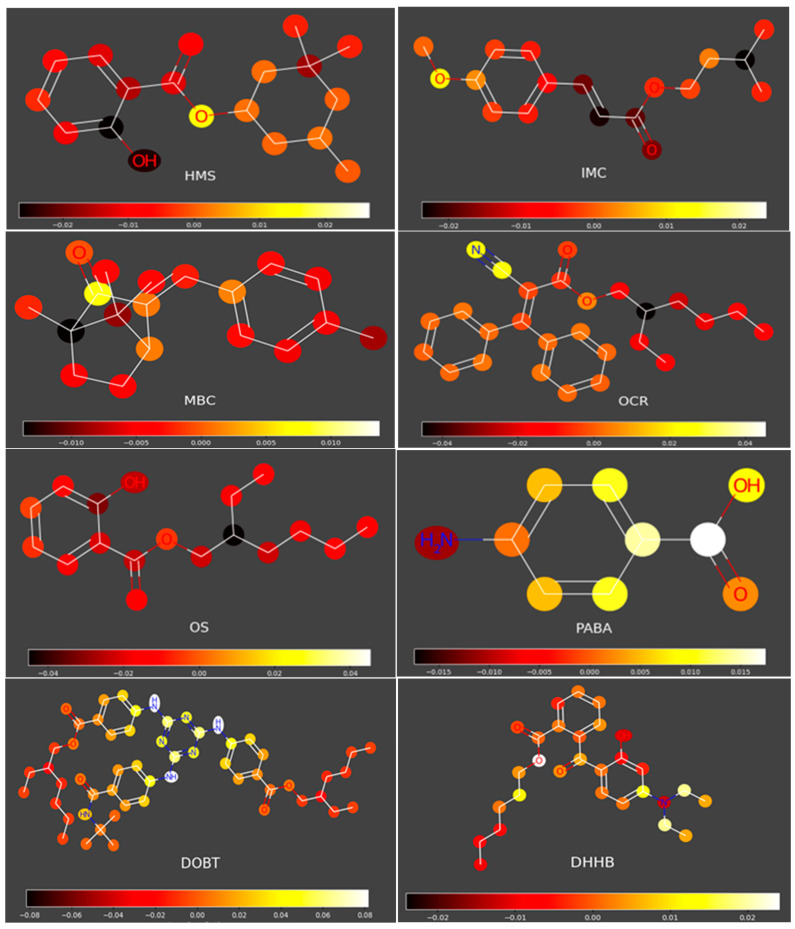
Atomic contributions to p***IC***_50_—organic sunscreens.

According to our brief analysis of atomic contributions (Figure 6), the atoms/groups that reduce p***IC***_50_ are long aliphatic chains, phenolic OH, NH_2_ in aromatic amines, and SO_3_H groups; the atoms/groups that act in the opposite direction are N in benzimidazole and triazine rings, NH groups adjacent to them, and COOH groups.

Human butyrylcholinesterase (BChE) has a single catalytic active site located at the bottom of a ~20 Å–deep active-site gorge within the core of the enzyme. The subsites of BChE are as follows [110]:

○ Catalytic triad—Ser198, His438, Glu325;
○ Choline binding site—Trp82;
○ Peripheral anionic site—Asp70, Tyr332;
○ Oxyanion hole—Gly116, Gly117, Ala199;
○ Acyl pocket—Leu286, Val288.

**Figure 7:**
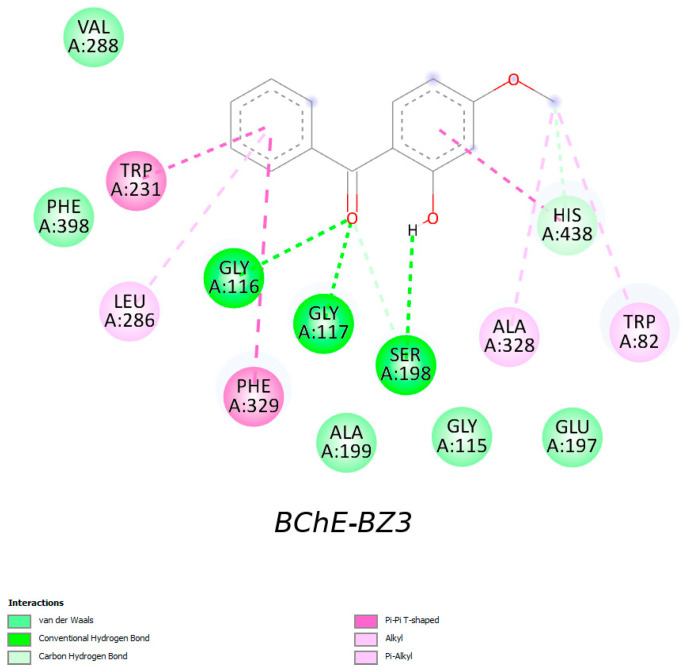
Interactions of BChE with benzophenone-3.

According to our brief molecular docking study, the sunscreens primarily interact with His438, Ser198, Trp82, Phe329, and Gly116 (details provided in the Supplementary Materials). For example, benzophenone-3 (permitted for use in most countries worldwide despite its good dermal absorption and several established or possible health and environmental issues) engages in interactions with amino acid residues in BChE’s catalytic triad, oxyanion hole, choline binding site, and acyl pocket (Figure 7):

- Conventional H-bonds (C=O with Gly116 and Gly117; OH with Ser198);
- Carbon-H bonds (C=O with Ser198 and CH_3_ with His438);
- π-π (benzene rings with Trp231, Phe329 and His438);
- Alkyl (CH_3_ with Ala328 and Trp82);
- π-alkyl (benzene ring with Leu286);
- Van der Waals (Val288, Gly115, Glu197, Ala199, and Phe392).

Van der Waals interactions are general atom-to-atom attractions; alkyl/hydrophobic contacts are a subset that emphasizes the burial of nonpolar groups; carbon-hydrogen bonds are weak hydrogen bonds that are not captured by classical H-bond definitions. They are reported separately to reflect both the physical distinctions among them and practical considerations for drug design [111,112]. Given the systemic availability of BZ3, its interactions with BChE in vivo are not unlikely.

In the case of SO_3_H (PBSA), unfavorable negative-negative interactions between this group and ASP70, a residue at the peripheral anionic site of BChE, are possible. This may be why PBSA is a weaker inhibitor than PABA (the COOH group at physiological pH exhibits no such interaction). The opposing contributions of carboxylate and sulfonate groups to BChE binding may stem from differences in size, hydration, and desolvation properties. Therefore, sulfonic acid ligands such as PBSA or BZ-4 may be less compatible with the BChE-binding gorge than carboxylic acids.

## 3. Materials and Methods

### 3.1. Reference Compounds

The experimental ***IC***_50_ (nM) values for 121 compounds used to create QSAR models were taken from [103,113]. The compounds used to generate the MLR and SVR models were randomly assigned to one of the two sets: a training set (n = 100) and a test set (n = 21) using XLSTAT v. 2025.2.0 from Lumivero (Denver, CO, USA); in ANN models, the training/test/validation sets were randomly selected using Statistica v. 13.3.

### 3.2. Calculated Molecular Descriptors and Membrane Permeability Data

Physico-chemical and ADMET properties were calculated using ADMETLab3.0 software, with SMILES strings generated in ACDLabs ChemSketch v.2023.1.2 as input. The physico-chemical descriptors considered relevant in this study are: molecular weight (***MW***); ***MW***/***Vol*** (***Dense***); topological polar surface area (***TPSA***); count of hydrogen bond acceptors (***nHA***); count of hydrogen bond donors (***nHD***); count of rotatable bonds (***nRot***); count of rings (***nRing***); number of atoms in the largest ring (***MaxRing***); count of non-carbon atoms (hydrogens included) (***nHet***); count of rigid bonds (***nRig***); ***nRot***/***nRig*** (***Flex***); logarithmic aqueous solubility (log***S***); logarithmic n-octanol/water partition coefficient (log***P***); logarithmic n-octanol/water distribution coefficient at pH = 7.4 (log***D***); and fraction of sp3-hybridized carbon atoms (***Fsp3***). ADMET properties considered in the study are: calculated permeabilities (***caco2***; ***MDCK***; ***PAMPA***); volume of distribution at steady state (***VDss***); plasma protein binding, % (***PPB***); and fraction unbound in plasma, % (***Fu***). Descriptor values for the reference compounds and organic sunscreens are provided in the Supplementary Materials.

### 3.3. Multiple Linear Regression (MLR) Models

Multiple linear regression models were generated in XLSTAT from Lumivero, using descriptors calculated by ADMETLab3.0, in “best subset selection” mode, with the number of independent variables set between 2 and 6 and the tolerance level set at 0.1 (it is assumed that two descriptors are collinear if the tolerance value between them, calculated as (1 − R^2^), is <0.1 [114]). The MLR models were validated using R^2^, R^2^_adj._, and Q^2^ metrics for the training set, and RMSE_pred_ (Root Mean Square Error of prediction) for the test set [115,116,117].

### 3.4. Artificial Neural Network (ANN) Models

Multilayer Perceptron (MLP) artificial neural networks (ANNs) were generated using Statistica v.13.3 (regression, Automated Network Search—ANS module, 500 networks to train, 50 networks to retain), based on the same set of independent variables as in Section 3.3. The neuron activation functions considered were: identity, logistic, hyperbolic tangent, and exponential. The BFGS (Broyden–Fletcher–Goldfarb–Shanno) algorithm was used to train the networks. The error function was the sum of squares (SOS). The ANN models were evaluated using correlation coefficients for the training, test, and validation sets. The importance of independent variables in the ANN models was evaluated using global sensitivity analysis (GSA), which quantifies each input variable’s importance by computing the sum of squared residuals for the model without that variable relative to the full model. When an input variable scores 1 or less in GSA, the network performs better without it. Detailed data on the five retained networks, including the correlation coefficients and the GSA results, are provided in the Supplementary Materials.

### 3.5. Support Vector Regression (SVR) Model

The Support Vector Regression model was generated in XLSTAT using the set of independent variables previously used in Section 3.3 and Section 3.4. The kernel functions considered initially were linear, quadratic, RBF, and sigmoid, with the linear kernel yielding the best results in terms of R^2^, Mean Absolute Error (MAE), and Mean Squared Error (MSE) for both the training and test sets.

### 3.6. Applicability Domain

The applicability domain of QSAR models was established using Principal Component Analysis of key physicochemical properties of the studied compounds (“Sunscreens”) and reference compounds (“Inhibitors”) described in Section 3.1. Principal Components (PCs) were calculated using XLSTAT (see Supplementary Materials). The 1st vs. 2nd PCs of the studied and reference compounds were plotted in a 2D coordinate system, and the area covering the chemical space of 99% of the reference compounds was indicated by an ellipse (Figure 4).

### 3.7. Analysis of Atomic Contributions Influencing pIC_50_

Hologram QSAR analysis of p***IC***_50_ was conducted using ChemMaster v.2 software, based on Morgan circular fingerprints with radius = 2 and the number of bits = 2048, pre-treated with PLS (6 components), with 5-fold cross-validation. The 121 reference compounds and the organic sunscreens investigated in this study were randomly assigned to a training set (75%) and a test set (25%).

### 3.8. Molecular Docking

Molecular docking was performed using MolModa 1.0.1 software from Durrant Labs (Pittsburgh, PA, USA) [118] with the structure of human BChE extracted as a PDB file from the RCSB Protein Data Bank (https://www.rcsb.org): PDB ID—4BDS (crystal structure of human butyrylcholinesterase in complex with tacrine) [103]. The settings for the drug-enzyme docking were as follows: sampling exhaustivity = 8; box center coordinates: x = 136.4; y = 115.0; z = 39.7; box size: 15 × 12 × 12 Å.

The enzyme structure (solvent and other small molecules removed) was protonated using a suitable MolModa option.

The ligands were uploaded as SMILES strings retrieved from the PubChem database. Their SMILES strings were converted to 3D structures with maximum force-field optimization, protonated at pH 7.4 using the built-in Open Babel [119] functionality, and the resulting 3D structures were optimized again using the maximum force-field optimization.

The protein–ligand interactions of the BChE-ligand complexes were visualized using Discovery Studio 2024 software (Biovia, San Diego, CA, USA) (Supplementary Materials).

## 4. Conclusions

Organic sunscreens have long been known to affect both human and animal health, but so far, the main issues studied in the context of organic sunscreens and their behavior in organisms have been absorption through the skin or the gastrointestinal tract, and interference with human/animal hormone systems. However, some studies have shown that organic sunscreens (e.g., MBC) can reduce AChE activity in zebrafish embryos in vivo and in mammalian cells in vitro [120]. EHDP and BMDM have also been found to inhibit AChE in crucian carp [121]; hence, AChE is an established biological target for organic sunscreens, and their interactions with animal or human cholinergic systems are not unlikely.

In this study, the focus was on another cholinesterase—BChE—and the ability of selected sunscreens to inhibit it was predicted using Multiple Linear Regression, Artificial Neural Network and Support Vector Regression models based on self-explanatory descriptors related to compounds’ drug-likeness and ADMET properties, such as the count of rotatable bonds (***nRot***), lipophilicity (log***D***, flexibility (***Flex***), fraction of sp^3^ carbon atoms (***F***_sp3_), caco-2 permeability (***caco2***) and plasma protein binding ability (***PPB***). The models developed herein, however, have some limitations—they may overestimate the inhibitory activity of very bulky, highly lipophilic molecules with many degrees of freedom (DOBT, ET) that fall outside the applicability domain defined by the properties of reference compounds.

It was established that some of the compounds investigated in this study are likely to inhibit BChE, and that their ***IC***_50_ values are comparable to those of established cholinesterase inhibitors (e.g., donepezil and rivastigmine). Given the risks associated with low BChE levels in the organism, such as altered drug metabolism, increased susceptibility to organophosphate poisoning, and metabolic issues, including impaired fat metabolism, it was concluded that organic sunscreens should be investigated further in the context of their potential interactions with both the neurotransmission and metabolic functions of the cholinergic system.

## Figures and Tables

**Table 2:** Key descriptors and the p***IC***_50_ values calculated using MLR, ANN1 and SVR models (Mean^(1)^—calculated for MLR, ANN1 and SVR values; Mean^(2)^—ANN2 do ANN5 values also included; the highest values of ***IC***_50_ are bolded).

	*nRot*	*Flex*	*Fsp3*	*logD*	*caco2*	*PPB*	MLR	ANN1	SVR	Mean^(1)^	Mean^(2)^
**BMDM**	6	0.429	0.300	4.08	−4.70	96.29	4.95	4.52	4.83	4.77	4.82
**BP-3**	3	0.231	0.071	3.42	−4.86	97.81	4.19	4.15	4.09	4.14	4.18
**DHHB**	12	0.857	0.417	4.26	−4.75	98.13	**5.57**	**4.97**	**5.37**	**5.30**	**5.27**
**PABA**	1	0.143	0.000	1.00	−5.24	43.55	4.48	5.10	4.66	4.75	4.87
**EHDP**	9	1.286	0.588	3.89	−4.90	98.46	1.30	3.27	1.42	2.00	2.77
**Et-PABA**	3	0.429	0.222	1.97	−5.12	74.52	2.79	3.64	2.90	3.11	3.45
**PBSA**	2	0.111	0.000	1.62	−5.59	98.51	2.90	3.64	2.80	3.11	3.54
**MBC**	1	0.063	0.500	3.85	−4.57	95.07	3.76	3.57	3.78	3.70	3.64
**EHMC**	10	1.250	0.500	3.86	−4.90	98.60	2.07	3.34	2.12	2.51	3.05
**IMC**	7	0.875	0.400	3.59	−4.80	96.20	2.47	3.39	2.50	2.79	3.20
**OCR**	10	0.667	0.333	4.52	−4.94	99.28	**6.13**	**5.78**	**5.91**	**5.94**	**5.96**
**ET**	30	1.111	0.500	5.09	−4.99	100.70	**13.82**	**14.82**	**12.94**	**13.86**	**10.70**
**OS**	8	1.143	0.533	3.53	−4.89	98.07	1.24	3.27	1.34	1.95	2.75
**HMS**	3	0.231	0.562	3.58	−4.87	98.30	3.50	3.51	3.49	3.50	3.57
**DOBT**	25	0.926	0.455	4.72	−5.06	99.40	**12.14**	**13.28**	**11.40**	**12.27**	**9.90**
**BZ-4**	4	0.267	0.071	1.87	−5.49	98.90	3.12	3.70	3.00	3.27	3.65

**Table 3:** Correlation matrix (R), n = 121.

	*Flex*	log*D*	*PPB*	*caco2*	*nRot*	*Fsp3*	p*IC*_50_ Exp
***Flex***	1.000	0.203	0.022	0.056	0.864	0.537	−0.026
log***D***	0.203	1.000	0.648	−0.003	0.274	−0.084	0.446
***PPB***	0.022	0.648	1.000	−0.081	0.119	−0.393	0.119
***caco2***	0.056	−0.003	−0.081	1.000	−0.047	0.220	−0.360
***nRot***	0.864	0.274	0.119	−0.047	1.000	0.463	0.297
***Fsp3***	0.537	−0.084	−0.393	0.220	0.463	1.000	−0.154
p***IC***_50_ exp	−0.026	0.446	0.119	−0.360	0.297	−0.154	1.000

**Table 4:** SVR performance metrics (p***IC***_50_).

Statistics	Training Set	Validation Set
**MSE**	0.291	0.498
**R^2^**	0.710	0.661
**MAE**	0.432	0.495

## Data Availability

The original contributions presented in this study are included in the article and Supplementary Materials. Further inquiries can be directed to the corresponding authors.

## Supplementary Materials

The following supporting information can be downloaded at: https://www.mdpi.com/article/10.3390/molecules31101656/s1.
